# Complications Following MitraClip Implantation

**DOI:** 10.1007/s11886-021-01553-9

**Published:** 2021-08-13

**Authors:** Katharina Schnitzler, Michaela Hell, Martin Geyer, Felix Kreidel, Thomas Münzel, Ralph Stephan von Bardeleben

**Affiliations:** Department of Cardiology, University Medical Center of the Johannes Gutenberg-University Mainz, Langenbeckstraße 1, 55131 Mainz, Germany

**Keywords:** Mitral regurgitation, MitraClip, Transcatheter edge-to-edge repair (TEER), Transcatheter mitral valve repair (TMVR), Complications

## Abstract

**Purpose of Review:**

To provide a detailed overview of complications associated with MitraClip therapy and its development over time with the aim to alert physicians for early recognition of complications and to offer treatment strategies for each complication, if possible.

**Recent Findings:**

The MitraClip system (MC) is the leading transcatheter technique to treat mitral regurgitation (MR) and has been established as a safe procedure with very low adverse event rates compared to mitral surgery at intermediate to high risk or in secondary MR. Lately, the fourth MC generation has been launched with novel technical features to facilitate device handling, decrease complication rates, and allow the treatment of even complex lesions.

**Summary:**

Although the complication rate is low, adverse events are associated with increased morbidity and mortality. The most common complications are bleeding, acute kidney failure, procedure-induced mitral stenosis, and an iatrogenic atrial septal defect with unknown clinical impact.

**Supplementary Information:**

The online version contains supplementary material available at 10.1007/s11886-021-01553-9.

## Introduction

Mitral regurgitation (MR) is the leading heart valve disease and associated with high mortality and morbidity [1–3]. To address the many untreated patients with severe MR and high surgical risk, the transcatheter edge-to-edge repair (TEER) with MitraClip® (MC) (Abbott, Menlo Park, CA, USA) was introduced as a first in-human repair option in 2003 [4–8]. Derived from the surgical Alfieri-stitch approach, MC is the leading transcatheter mitral valve repair (TMVR) technique with over 110.000 devices implanted worldwide and has been approved for primary and secondary MR in Europe and the USA [9, 10]. The latest valvular heart disease guidelines recommend TEER for treatment of symptomatic patients with secondary MR under optimal guideline-directed treatment of heart failure and ideally fulfilling COAPT-like criteria (IIa LOE B recommendation). These patients should be first-line evaluated for TEER and secondarily for surgical replacement (IIb LOE B recommendation). For primary MR in severely symptomatic patients with high or prohibitive surgical risk of the elderly, TEER is now recommended as an addition to surgical repair if anatomy is favorable for TMVR [11••].

For patients with high surgical risk, TEER has been established as a safe procedure with very low adverse event rates compared to mitral surgery. Though, if complications occur, this may lead to a poorer outcome with increased mortality, and all heart team members, cardiologists, heart surgeons, imagers, anesthesiologists, heart failure and rhythm specialists, and geriatricians alike have to be aware of MC-associated complications [12–14]. This review provides a comprehensive overview of the complications associated with MC therapy in relation to the different device generations. Although there have been prior reports summarizing complications after TEER, this is, to our best knowledge, the first report including the novel and improved MC Generation 4 [15•].

## Evolution of MC

**Fig. 1:**
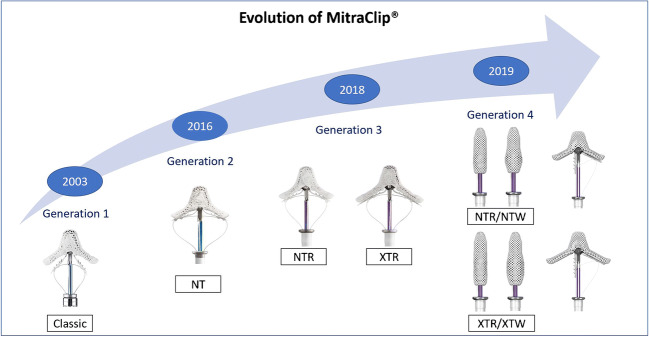
Evolution of MitraClip. This figure demonstrates the four MitraClip generations. (MitraClip TM is a trademark of Abbott or its related companies. Reproduced with permission of Abbott, © 2021. All rights reserved)

After the first in-human implantation in 2003, the MC system has undergone a continuous technical improvement to facilitate device handling and decrease complication rates [8]. Figure 1 visualizes the development of four MC generations, and Table 1 summarizes the technical advances made in each generation.

**Table 1:** Technical enhancements of MitraClip

	Generation 1	Generation 2	Generation 3		Generation 4			
	Classic	NT	NTR	XTR	NT	NTW	XT	XTW
Technical details								
Arm length	9 mm	9 mm	9 mm	12 mm	9 mm	9 mm	12 mm	12 mm
Arm width	4 mm	5 mm	5 mm	5 mm	4 mm	6 mm	4 mm	6 mm
Fictional elements	4	4	4	6	4	4	6	6
Catheter outer diameter	24 F	24 F	24 F	24 F	25 F	25 F	25 F	25 F
Novel device features added to reduce complications	• Change from suture to gripper plate with 4 frictional elements and stainless arms • Z lock to L lock	• Gripper material change from Elgiloy to Nitinol • Steerable sleeve enhancements • Gripper lowering to leaflet improved	• 2 sizes available for TEER (elongated arms 9 to 12 mm XTR with 2 more frictional elements) • Improved catheter delivery system with a stiffer and 1.5-cm longer shaft Ability to open and close arms without a locking device		• 4 sizes available for TEER (with 4 to 6 mm, arm length spans 15 to 18 mm), pressure line • Dual or separate independent grasping • Continuous left atrial pressure monitoring • Precise and predictable steering Simplified system preparation and deployment			

*TEER*, transcatheter edge-to-edge repair

The newest MC “Generation 4” or “G4,” launched in 2019, with four different sizes and a 50% greater grasping area offers the implanter more flexibility to treat even complex lesions while reducing leaflet stress. “G4” features a novel side-dependent grasping and real-time left atrial pressure monitoring. Furthermore, preparation is simplified and steering more precise [16••, 17, 18].

An alternative TEER device to MC on the European market is the PASCAL system (Edwards Lifesciences, Irvine, CA, USA), which received CE mark in 2019 and has achieved an implant experience of more than 1,500 devices in humans. The main structural difference to the MC is a 10-mm central spacer, which is supposed to fill the regurgitation orifice. The new PASCAL Ace with a 6-mm width offers an enhanced effective arm length of 10 instead of 9 mm compared to the traditional PASCAL [17, 19]. The ongoing CLASP IID/IIF Pivotal Clinical Trial (NCT 03706833) will compare the performance of the PASCAL with the MC system.

Continuous improvement of the MC system allows effective treatment in even complex anatomies; however, crossing boundaries can lead to increased procedural and device failure, and it needs an experienced interventionalist to choose the right clip to meet the individual anatomy. With the rise of novel transcatheter mitral valve replacements, MC therapy will have to assert itself in terms of successful treatment and procedural safety, especially in complex anatomies [16••, 17].

## Complications

After the promising results of EVEREST I trial in 2005, multiple randomized, controlled trials and retrospective registries confirmed the low complication rates with high procedural performance for MC therapy [4, 5, 7, 13, 20, 21•, 22•, 23, 24••, 25, 26]. Technical developments and growing experience with MC decreased the major adverse event rate from 15% in 2005 to <3.5% in 2020, even though more complex lesions have been addressed lately [4, 17, 27]. Patients suffering from MC-associated complications are typically older, female, and in a poorer health status. Complications lead significantly to more re-interventions, an extended hospital stay, and increased mortality compared to a noncomplicated course [14].

**Fig. 2:**
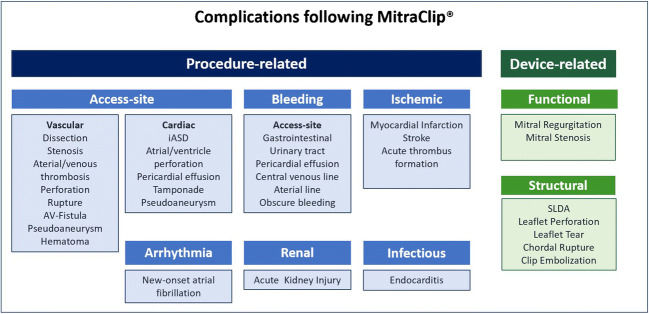
Complications following MitraClip implantation. *iASD*, iatrogenic atrial septal defect; *SLDA*, single leaflet device attachment

The Mitral Valve Academic Research Consortium (MVARC) standardized the endpoint and complications definitions for TMVR in 2015 [28]. Nevertheless, definitions of complications still vary in literature. MC-associated complications can be considered as procedural- or device-related (Fig. 2) and will be discussed in detail in the following sections [15•].

### Procedure-Related Complications

Procedure-related complications mainly result from a large-lumen access and the transseptal puncture.

#### Death and Need for Resuscitation

Although most people treated with MC are high-risk patients, real-world data show a very low intraprocedural (TRAMI register 0.1%, Praz et al. 0%, Chakravarty et al. 0%) and in-hospital mortality (TRAMI 2.4%, TCVT 2.9%, German nationwide sample 3.6%, TVT 2.7%, NIS database 2.0%) [7, 12, 16••, 21•, 22•, 23, 29••]. Several studies noted a decline of in-hospital mortality over time (Elbaldawi et al.: 2012: 3.6%; 2016: 1.6%, p=0.06, Geyer et al. 2010/2011: 9.1%, 2017/2018: 0.6% p =0.001) [12, 30•]. The latest studies with the XTR/NTR and “G4” reported an even lower in-hospital mortality (XTR/NTR 0.9%, G4 0%) [16••, 29••]. Predictors of in-hospital mortality include severe heart failure, chronic kidney disease, male gender, tricuspid regurgitation and baseline pulmonary hypertension, blood transfusions due to anemia, stroke, endocarditis, pulmonary embolism, and pericardial effusion [12, 21•, 30•]. Cardiac arrest and in-hospital resuscitation are rare events (TRAMI 0.8%, ACCESS-EU 1.1%, NIS data 1.4%) with a significant decrease from 2012 to 2016 [5, 12, 14].

#### Access Site Complications

MVARC categorizes access-related complications in vascular and cardiac complications. The major events are associated with death or severe sequelae [28].

##### Vascular Complications

The large caliber 24-F catheter and the proximity of the femoral vein to the artery can cause severe vascular complications. Studies report rates of 1.4–4% for major [14, 22•, 29••, 31] and 2.7–3.8% for minor vascular complications [12, 14, 22•] without changes over time. Different access closure strategies, e.g., Z-Suture vs. ProGlide® (Abbott Vascular Inc., Santa Clara, California), did not show a difference in terms of safety [31, 32]. Preprocedural controlled hydration and ultrasound-guided venous puncture are small measures, which may help to reduce vascular complications. The “G4” contains a slightly larger-sized catheter (25F); the effect on vascular complication rates will have to be evaluated in upcoming studies.

##### Cardiac Complications

Pericardial effusion, caused by erroneous transseptal puncture or the clip-delivery system before or after clip deployment, and the persistence of iatrogenic atrial septal defect (iASD) embody the main access-related cardiac complications.

Transesophageal echocardiography (TEE) guiding supports a controlled and safe transseptal puncture with the aim of a high and posterior position for best access to the mitral valve. Pericardial effusion or tamponade are rare complications (0–0.5%) with a downwards trend over the years, likely to be related to higher implanter experience and consequent use of 3D-TEE guiding [5, 12, 14, 21•, 23, 29••]. Ultrasound-guided transseptal puncture is generally safe; nonetheless, it can be challenging in cases with a hypermobile septum, postsurgical or post-interventional closed iASD, or when a very small fossa ovalis requires puncture of the muscular part of the septum. Treatment of these cases should be reserved for experienced centers.

The rate of persistent iASD is 57, 50, and 25% after 1, 6, and 12 months post procedure suggesting a spontaneous closure is a long-term clinical course [33–36]. An elevated left atrial pressure after clip release correlates with iASD persistence [35]. Its clinical impact is controversially discussed: On the one hand, a poorer outcome of patients with iASD after MC therapy with increased mortality and rehospitalization rate has been reported [33, 34, 37, 38]. Additionally, iASD closure leads to a favorable volume shift from the right to the left ventricle with an increase in cardiac index and release of heart failure symptoms in symptomatic patients after TEER [39]. On the other hand, improvements in symptoms and hemodynamic parameters for intra-atrial shunt devices in patients with preserved ejection fraction have been demonstrated [40, 41]. Further, with an immediate reduction of left atrial pressure after removal of the MC catheter out of the left atrium at the end of MC procedures, Hoffmann et al. reported a positive effect of the iASD to the hemodynamic benefits of TEER [42]. The ongoing MITHRAS trial might enlighten the role of iASD after TMVR [39].

#### Bleeding Complications

Given the high coincidence of MR with other cardiac diseases, e.g., atrial fibrillation (AF), patients treated with TEER frequently already have an indication for anticoagulation or dual antiplatelet therapy (DAPT) [43]. To prevent thromboembolic complications, heparin administration interprocedurally is required but in turn raises the risk of bleeding. Further, the periprocedural TEE guiding may cause gastrointestinal injury, like esophageal perforation, laceration, or gastrointestinal bleeding [44]. Hence, it is not surprising that bleeding is one of the main adverse events after TEER. Severe bleeding acquiring blood transfusion occurs in 0–17% [9, 13, 14, 16••, 21•, 31, 43, 45] and has been found to be an independent predictor for in-hospital mortality [21•]. Recent studies with third and fourth MC generations showed decreased numbers of severe bleeding [16••, 29••]. Interestingly, despite the large-lumen catheter, less than 50% of bleedings are access-related. A high number of bleeding is from gastrointestinal origin. Other bleedings originate from the urinary tract, pericardial effusion, central venous catheter, cerebral or skin lesions, and arterial line or are obscure [43, 45]. Bleeding history, chronic kidney, and coronary artery disease are independent risk factors for bleeding complications following TEER [43, 45].

The balance of anticoagulation to avoid thromboembolic events and bleeding complications is challenging, and no guidelines exist for the optimal postprocedural treatment. For the EVEREST trials, antihemostatic naïve patients received a DAPT with aspirin 325mg for 6–12 months and clopidogrel 75mg for 1 month [13]. In Europe, a shortened therapy with aspirin 100mg for 3 months and clopidogrel 75mg for 1 month is common [46]. A significant lower thromboembolic rate was reported for a combined therapy of apixaban 2,5mg and aspirin 100mg following the first postprocedural 30 days compared to DAPT in a cohort with sinus rhythm. However, with 0% bleeding complications, this study does not represent real-world data [47]. Patients with indication for anticoagulation often remain on their initial anticoagulation therapy without therapy modification.

#### Thromboembolic Events

Using a large caliber catheter in the venous system, thrombo- or air-embolic complications deserve serious consideration with responsible handling of the catheter, constant flushing, precise device preparation, and adequate anticoagulation [15•]. Overall, major adverse cardiac and cerebrovascular events occurred in real-world data in 3–7% [14, 21•]. The in-hospital myocardial infarction rate ranges 0–3% [5, 12, 14, 21•, 22•, 25]. The incidence of a postprocedural stroke is 0–1% [5, 12, 14, 21•, 22•, 23, 29••]. Despite the risk of paradox embolism through an iASD, a meta-analysis found no difference in stroke rates in patients after MC therapy compared to patients on optimal medical therapy. The postprocedural stroke rate tends to be decreased compared to surgical mitral therapy (p=0.19), which might be due to a higher incidence of de novo atrial fibrillation in surgical patients [48•]. On the other hand, a diffusion MRI study reported an incidence of 86% new silent cerebral lesions after TEER in a cohort of 27 patients with a significant reduction in neurocognitive function tests if >3 cerebral lesions were present. Dementia or cognitive impairment may occur in the further clinical course after silent cerebral embolisms [49]. Though clinically noticeable stroke rates are low, this important topic deserves further investigations including the potential benefit of embolic protection systems.

Despite intraprocedural heparin administration, there is the risk of acute thrombus formation at the thrombogenic material of the catheters [49, 50]. Pregowski et al. found 9 out of 100 patients with acute thrombus formation at the transseptal needle, sheath, guiding catheter, or at the clip itself, while larger registries reported low rates of <0.5% (TRAMI registry 0.1%, Sorajja et al. 0%) [14, 22•, 50–52]. In these cases, a “wait and see” strategy with readministration of heparin, thrombus aspiration, or low-dose thrombolysis must be carefully weighed against each other [50–52].

#### De Novo Atrial Fibrillation

Few data describe the incidence of de novo AF after TEER. High rates (11.7%) of newly diagnosed AF were described at the Pilot European Sentinel Registry, whereas lower rates of 1.2% were found in the GRASP registry [23, 53]. A meta-analysis of low cohort number studies showed an incidence of 2.4% of de novo AF after MC [48•]. The clinical impact of de novo AF after TEER will have to be further investigated.

#### Acute Kidney Injury

TEER-related acute kidney injury (AKI) occurs in up to 18% and is an independent risk factor for 1-year mortality [5, 12, 14, 29••, 53, 54]. Importantly, MC therapy does not require any contrast agent compared to other structural heart interventions. Factors likely to increase the risk of AKI are a shortfall of mean arterial pressure during general anesthesia or systemic inflammatory response due to vascular access or artificial material in the bloodstream [54]. Baseline renal function, serum proBNP-levels, HbA1c, diuretic usage, and elevated right atrial pressures also correlated with AKI [54]. An optimal fluid balance, blood sugar control, periprocedural pause of nephrotoxic medication, and cautious blood pressure monitoring might decrease the risk of AKI after TEER.

#### Endocarditis

Mitral valve endocarditis after TEER is a rare event (0–2.6%) with only few cases reported [55, 56].

[14, 57–60]. Similarly, a low incidence of endocarditis was reported for the surgical double-orifice technique (0.7%) [61]. Possibly, the small amount of artificial material in the bloodstream and the fibrous encapsulation of the clips explain the low number of valve infections after TEER. The diagnosis of endocarditis in TEER patients is challenging, which may lead to underdiagnosis [62]. Most endocarditides appear in the first year after MC (75%), and in addition to an optimal antibiotic therapy, a surgical valve replacement is unavoidable in most cases (70%). The reported in-hospital mortality of 40% is very high, irrespectively, if antibiotic-only or a combined therapy was applied, so awareness and prophylaxis of this rare, but severe complication are crucial [55].

### Device-Related Complications

Device-related complications include functional (e.g., persistent MR, mitral stenosis (MS)) and structural device failure (e.g., clip detachment with possible clip embolization, injury of leaflets, or subvalvular apparatus).

#### Functional Device Failure

##### Persistent Mitral Regurgitation

Persistent MR is an important prognostic factor for mortality and rehospitalization after MC [5, 22•, 63]. While early studies reported a MR reduction <2+ at a discharge of less than 80% (Everest I 64%, EVEREST II 77%), the latest studies with third and fourth MC generation achieved a MR reduction <2+ of >95% [13, 16••, 20, 29••, 64]. Praz et al. described a remarkable increase of patients with trace or mild residual MR of 93% for the “G4,” with only 3.5% of patients presenting with a persistent MR >2+ at 30 days [17]. Both new features of “G4,” wider clip arms and independent grasping, are thought to be responsible for this excellent improvement of procedural results.

MR reduction depends on the implanters experience to identify an optimal strategy for each pathology and to select the most favorable clip size [27, 65]. Experienced compared to inexperienced implanters perform significantly better in reduction to mild MR [27]. The wider clip versions (NTW/XTW) were frequently used for patients with broader MR jets and in cases when the implanter intended to implant only one clip. Primary MR with large prolapse is rather treated with the XT/XTW [16••, 66••].

##### Mitral Stenosis

Clip-associated MS is related to worse long-term outcomes and higher mortality [28, 63]. MVARC defines a postprocedural MS, if the mean transvalvular pressure gradient (MPG) is >5 mmHg. Unfortunately, most studies provide only inadequate or even missing information regarding MS rates and their definition. No mitral stenosis was seen at the EVEREST II trial. In the TRAMI registry, <1% were observed [13, 14]. Other studies, defining a stenosis at MPG >5 mmHg, describe a high incidence of 25–35% [63, 67–69]. With a better mitral toolbox, a recent study on “G4” with the alternative treatment option of a transcatheter valve replacement in TEER-suboptimal patients reported a lower rate of MS (15%) at the expense of a higher implantation failure rate [16••].

Biaggi et al. detected an elevated MPG >5 mmHg measured with continuous-wave Doppler echocardiography as the best intraprocedural predictor to indicate MS at discharge [67]. Due to the double-orifice technique, the MPG increased significantly at discharge compared to baseline and remained constant in the follow-ups over 2 years, indicating a low, late MS rate [23, 29••, 67, 70]. Neuss et al. observed an increased mortality for patients with MPG >4.4 mmHg measured echocardiographically and 5 mmHg measured invasively after clip implantation.

The most important preprocedural risk factor for a relevant postprocedural MS is a mitral valve area <4 cm^2^ measured by 3D-TEE [29••, 63, 69]. As different valve anatomies trigger the use of different MC sizes, there is no evidence that MC of greater size or width or even a higher number of MC creates higher degrees of MS. One study has reported higher transvalvular gradients in patients treated with a combination of MC NTR and XTR, a finding that is most probably explained by smaller anatomies as in patients receiving >1 MC XTR [16••, 66••]. Given the evidence of a worse outcome with clip-induced MS, high gradients have to be avoided, and the MC should not be deployed unless acceptable gradients can be accomplished [63].

#### Structural Device Failure

##### Single Leaflet Device Attachment

Single leaflet device attachment (SLDA), also known as a single leaflet or partial clip detachment, describes the complete loss of clip connection to one leaflet (videos 1, 2, 3). It is the most common structural device failure after TEER and occurs more frequently in complex lesions [5, 16••, 22•, 29••]. Most clips detach acutely (during the procedure) or subacutely (first days after the procedure), while late SLDA is infrequent [5, 15•]. It is assumed that SLDA mainly follows insufficient leaflet grasping, while SLDA after adequate grasping is typically caused by leaflet tear or perforation [5, 71]. A biomechanical model confirms that an asymmetrical or uncomplete grasp, both implying fewer tissues caught, increases leaflet stress [72]. The fibrous encapsulation of the clip over time might stabilize the leaflet insertion, so late SLDA is a rare finding. There has been a continuous reduction in SLDA over years, which likely reflects the implanter’s learning curve and advances in the clip systems: EVEREST I 11.0%, EVEREST II 5.1%, ACCESS-EU 4.8%, TRAMI 2.0%, TVT 1.5%, Praz et al. 4.0%, Mitra EXPAND 1.9%, and “G4” 1.7% [5, 13, 14, 16••, 20, 22•, 29••, 66••]. The high rate of SLDA at the first insights of the EXPAND data by Praz et al. with four out of 107 cases and two cases of leaflet tearing led to the hypothesis of an increased risk for XTR to injure the leaflets due to the longer arms with a higher force on the leaflet per area [17, 29••]. Therefore, the MC XTR is discussed to be used with special care. In particular, the tension on the leaflets should be avoided, especially in short or fragile leaflets or in valves with significant annular calcification [29••].

In our experience, optimal TEE imaging significantly reduces the risk of suboptimal leaflet insertion and SLDA. Three major steps in periprocedural imaging ensure an optimal leaflet insertion: First, a precise rotation check of the device by 3D-TEE to avoid asymmetric grasping; second, a meticulous control of the whole grasping process by biplane TEE guiding to guarantee a deep insertion of both leaflets; and third, a precise, but quick confirmation of sufficient leaflet insertion without risking a possible alteration of the tissue bridge by a too lengthy evaluation, while the clip is still attached to the delivery catheter.

SLDA can cause recurrence or even aggravation of MR and thus comprises a potentially serious adverse event. If technically possible, re-treatment of MC implantation can be undergone [5, 22•]. The new features of MC “G4,” i.e., wider clip arms that reduce the force on the leaflets and independent grasping, are designed both to improve leaflet insertion and reduce the rate of SLDA. Early data support this hypothesis [16••].

##### Clip Embolization

Clip embolization after complete detachment of both leaflets is a great rarity. Only two studies, the TCVT and TVT, describe clip embolization in 0.7% and 0.1% [22•, 23]. In the rare case reports, one clip embolized in the right axillar artery without any symptoms and required no further treatment [73]. Another clip was localized in the renal artery and one clip, detached due to endocarditis, stuck in the apex of the left ventricle without a possibility for surgical removal [62, 74].

##### Leaflet Injury/Chorda Rupture

Grasping leaflets can potentially injure the leaflet tissue, especially in thinned leaflets or in valves with annular sclerosis reducing the flexibility of annulus and leaflet base. The literature describes an incidence of leaflet injury of 0–2% [16••, 29••, 66••]. Valve injury is frequently found in patients with persistent MR after TEER: In 29% of persistent primary MR, one leaflet was torn and, in 18% of secondary MR patients, had a new prolapse or flail either through spontaneous chorda rupture or iatrogenic damage [35].

**Fig. 3:**
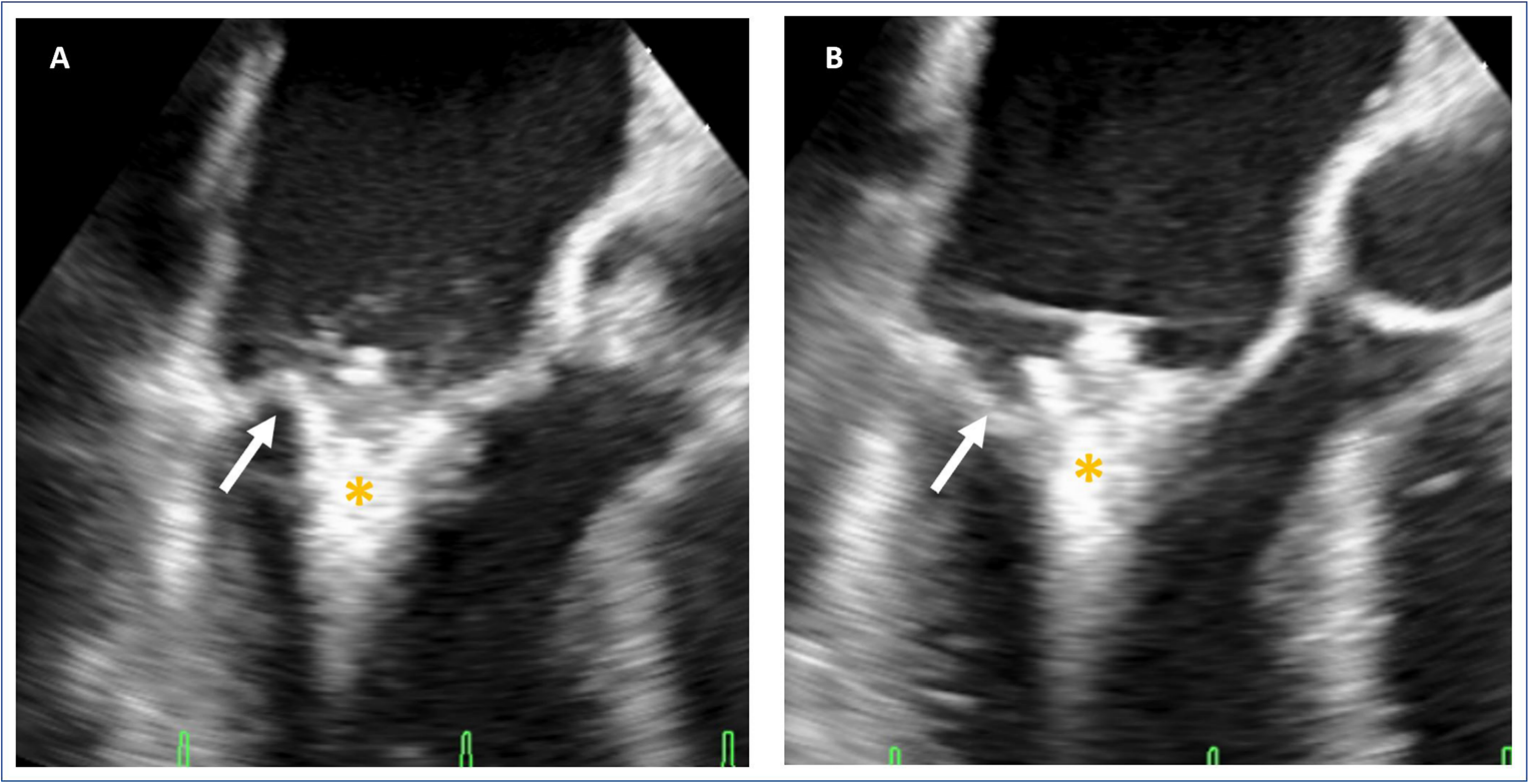
Perforation. Presentation of an intraprocedural leaflet perforation during grasping. **A** Transesophageal commissural view demonstrating the grasp of the leaflet showing the posterior leaflet (*arrow*) still on the clip (*). **B** Transesophageal commissural view showing the perforated posterior leaflet (*arrow*). *MitraClip

Mechanisms of injury are either leaflet perforation (Fig. 3, video 4) by the end of the clip arm or leaflet tear parallel to the clip arm. To prevent leaflet damage, excessive tension on the leaflet has to be avoided. In extended reach MC XT, where the longer lever of the clip arm is more prone to cause leaflet injury, an important measure of caution is a slight advancement of the clip in ventricular direction during clip closure [29••]. Treatment of leaflet damage is difficult as further grasping attempts may aggravate the problem. An effective bailout maneuver can be to grasp the injured leaflet further at the base either by a different device position or with a device with longer clip arms. Another mechanism of valve injury is the entrapment of the MC in leaflets or subvalvular structures with potential chordal rupture. Also, the new grasping options of sequential closure and consecutive movements with rotation may potentially benefit efficacy but could harm the integrity of the leaflet structure especially in the large annulus.

### Rare Complications

Rarities after TEER are subacute hemolytic anemia, post-cardiac injury syndrome, and interatrial septal dissection [75–78]. Two cases were found with hemolytic anemia following MC implantation. The first cause for hemolysis was a persistent severe MR after clipping a commissural prolapse at the posteromedial commissure, which was treated conservatively with blood transfusion and vitamin supplementation [76]. The other case was caused by mitral stenosis [75].

Post-cardiac injury syndrome was diagnosed in a patient with fatigue, elevated inflammation markers, and pericardial and pleural effusions following few days after TEER. Treatment with aspirin and colchicine reduced symptoms and laboratory parameters [78]. Interatrial septal dissection rarely occurs after mitral valve surgery, where a false lumen is formed between the mitral valve annular area and atrial septum or free atrial wall. One case was reported after TEER, which was treated conservatively [77].

### Options of Therapy After Failed Clip

The rapid increase of MC implantation and the treatment of more complex lesions increase the absolute number of MVARC-I device failures, even though TEER success rates now exceed 96% [16••, 21•]. Patients with persistent MR or SLDA with low transmitral gradient can be considered for a reclip procedure. However, elevated MPG or severe leaflet or chorda injury excludes patients from reclipping. As surgical treatment might only be an option for a few fitter patients, novel therapy with an electrosurgical detachment of the clip of one leaflet and transcatheter mitral valve implantation shows promising results. Lisko et al. presented a series of five cases, where the clip was detached of the anterior leaflet by electrosurgical laceration, leaving the clip to the posterior leaflet. Then, a mitral valve replacement with transapical access using the CE-marked Tendyne prosthesis (Tendyne Holdings, LLC, Roseville, Minnesota—a subsidiary of Abbott Vascular) was performed. Despite a high rate of life-threatening bleeding and hemothorax (both 40%), a procedural success rate of 100% and 30-day mortality of 0% are promising [79•]. More interventional tools are likely to appear that enable the heart team to replace repair by transcatheter beating heart replacement.

## Conclusion

TMVR using MC is a safe procedure with continuous device iterations improving the treatment of complex lesions. Though its safety has been demonstrated in several trials, complications increase morbidity and mortality. To reduce complications, implanters have to be aware of these adverse events and their risk factors and be familiar with their prophylaxis and bailout options, if needed. Latest data on MC “G4,” though still limited, indicate a reduction of complications due to the novel technical adjustments. Further enhancements and more experience with this device should potentially avoid future complications.t1

## Supplementary Information

ESM 1 . Development of an SLDA. Demonstrates the intraprocedural result after MitraClip implantation with implantation of one MitraClip “G4” XTW in mitral valve with 3D transesophageal glass view (left, atrial view; right, ventricular view). MitraClip was implanted because of primary severe mitral regurgitation (P2 flail leaflet). (AVI 25620 kb)

ESM 2 . Development of an SLDA. Presents a subacute single leaflet device attachment (SLDA) right before reclipping 5 days after initial clip procedure in 3D transesophageal glass view with an atrial and ventricular view. The clip is attached to the anterior leaflet. (AVI 12116 kb)

ESM 3 . Development of an SLDA. Shows a biplane-TEE loop presenting the result of the second MitraClip intervention. 2 XTW clips were implanted medial to the first clip. No stenosis occurred beside 3 clips in the mitral valve (mean transmitral gradient 2mmHg). The intervention resulted in mild persistent mitral regurgitation. (AVI 60077 kb)

ESM 4 . Intraprocedural perforation. Bi-plane transesophageal view during grasp of second MitraClip implantation resulting in perforation of posterior leaflet (the same patient as in Fig. 2). Besides multiple grasp attempts, no satisfying result was achieved, so the patient was immediately treated with surgical valve replacement. (AVI 137830 kb)

## Abbreviations

AF: atrial fibrillation
AKI: acute kidney injury
DAPT: dual antiplatelet therapy
iASD: iatrogenic atrial septum defect
MC: MitraClip
MPG: mean pressure gradient
MR: mitral regurgitation
MVARC: Mitral Valve Academic Research Consortium
SLDA: single leaflet device attachment
TAVI: transcatheter aortic valve implantation
TEE: transesophageal echocardiography
TEER: transcatheter edge-to-edge repair
TMVR: transcatheter mitral valve repair
